# ESUR: Opportunities for PSMA-PET/CT and whole-body MRI in advanced prostate cancer

**DOI:** 10.1007/s00330-025-12089-9

**Published:** 2025-11-05

**Authors:** Sungmin Woo, Luca Russo, Samuel J. Withey, Ailin Dehghanpour, Roberto García-Figueiras, Ivo G. Schoots, Giuseppe Petralia, Amish Lakhani, Tobias Penzkofer, Martina Pecoraro, Chen-Jiang Wu, Jochen Walz, Matthias Eiber, Wolfgang P. Fendler, Silke Gillessen, Raquel Perez-Lopez, Frédéric E. Lecouvet, Tara D. Barwick, Anwar R. Padhani

**Affiliations:** 1 Department of Radiology, NYU Grossman School of Medicine, NYU Langone Health, New York, NY USA; 2 Dipartimento Diagnostica per Immagini e Radioterapia Oncologica, Fondazione Policlinico Universitario A. Gemelli IRCCS, Rome, Italy; 3 Dipartimento Universitario di Scienze Radiologiche ed Ematologiche, Università Cattolica del Sacro Cuore, Rome, Italy; 4 Department of Radiology, Royal Marsden NHS Foundation Trust, London, UK; 5 Department of Radiological Sciences, Oncology and Pathology, Sapienza University of Rome, Rome, Italy; 6 Department of Experimental Medicine, Sapienza University of Rome, Rome, Italy; 7 Department of Radiology, Oncologic Imaging, Hospital Clínico Universitario de Santiago de Compostela, Santiago de Compostela, Spain; 8 Department of Radiology, The Netherlands Cancer Institute, Amsterdam, The Netherlands; 9 Department of Radiology and Nuclear Medicine, Erasmus University Medical Center, Rotterdam, The Netherlands; 10 Division of Radiology, IEO European Institute of Oncology, IRCCS, Milan, Italy; 11 Department of Oncology and Hemato-Oncology, University of Milan, Milan, Italy; 12 Department of Radiology, Mount Vernon Cancer Centre, Paul Strickland Scanner Centre, Northwood, UK; 13 Department of Radiology, Charité Universitätsmedizin Berlin, Berlin, Germany; 14 Berlin Institute of Health, Berlin, Germany; 15 Department of Radiology, The First Affiliated Hospital of Nanjing Medical University, Nanjing, Jiangsu China; 16 Department of Urology, Institut Paoli-Calmettes Cancer Centre, Marseille, France; 17 Department of Nuclear Medicine, Klinikum Rechts Der Isar, Technical University of Munich, Munich, Germany; 18 Department of Nuclear Medicine, University of Duisburg-Essen, Essen, Germany; 19 German Cancer Consortium (DKTK) University Hospital Essen, Essen, Germany; 20 Oncology Institute of Southern Switzerland, Ente Ospedaliero Cantonale, Bellinzona, Switzerland; 21 Faculty of Biosciences Università della Svizzera Italiana, Lugano, Switzerland; 22 Radiomics Group, Vall d’Hebron Institute of Oncology, Barcelona, Spain; 23 Department of Medical Imaging, Institut de Recherche Expérimentale et Clinique, Institut du Cancer Roi Albert II, Cliniques Universitaires Saint-Luc, Université Catholique de Louvain, Brussels, Belgium; 24 Department of Radiology, Charing Cross Hospital, Imperial College Health Care NHS Trust, London, UK

**Keywords:** Advanced prostate cancer, Prostate specific membrane antigen positron emission tomography, Whole-body magnetic resonance imaging, Metastasis

## Abstract

**Abstract:**

Prostate-specific membrane antigen (PSMA) positron emission tomography (PET) computed tomography (CT), and whole-body magnetic resonance imaging (WB-MRI) are superior to conventional CT and bone scan imaging for detecting metastatic disease in patients with prostate cancer. While these higher-accuracy imaging methods have already shown the potential to enhance patient outcomes, a thorough understanding of the relationship between the treatment landscape and disease volume on conventional imaging, as well as the prognostic significance of the prostate-specific antigen response, is crucial for determining how they can be more effectively incorporated. Prospective clinical trials are required to evaluate whether PSMA-PET/CT and WB-MRI can genuinely improve clinically relevant endpoints for patients through precise treatment adaptations. In this paper, we explore the specific opportunities of PSMA-PET/CT and WB-MRI as biomarkers in multiple clinical domains, including metastasis detection and staging, disease characterisation and aggressiveness assessments, biopsy target selection, impacts on treatment planning, evaluation of therapeutic response, and theranostics. We highlight the central research questions that require attention.

**Key Points:**

***Question*** *Can PSMA-PET/CT and WB-MRI, with their superior ability to detect metastases in prostate cancer, truly improve patient outcomes?*

***Findings*** *High-accuracy imaging improves metastasis detection, staging, assessment of disease aggressiveness, and enables more personalised treatment planning for advanced prostate cancer patients.*

***Clinical relevance*** *PSMA-PET/CT and WB-MRI have the potential to alter the management of men with advanced prostate cancer, but prospective clinical trials are needed to confirm benefits for survival or quality of life before recommending routine use.*

## Introduction

Advanced prostate cancer (APC) refers to locally advanced prostate cancer (PCa), metastatic hormone-sensitive prostate cancer (mHSPC), and metastatic castration-resistant prostate cancer (mCRPC) [1, 2]. APC has a range of outcomes, from aggressive pelvic-confined disease to rapid progression and death from metastases. In the coming decades, we can expect an increasing reliance on image-based diagnostic, prognostic, predictive, and response biomarkers to advance patient care. Imaging biomarkers will be integrated with molecular and clinical parameters to enhance risk-based diagnoses and inform therapy selection [3].

Key imaging methods include prostate-specific membrane antigen (PSMA) positron emission tomography (PET) computed tomography (CT) and whole-body magnetic resonance imaging (WB-MRI), which demonstrate superior diagnostic capabilities to detect metastatic disease compared with conventional imaging (bone scans (BS), CT, and regional MRI). Comparative studies show that PSMA-PET/CT outperforms WB-MRI in the detection of nodal and bone metastases [4–6]. As a result, PSMA-PET/CT has become an integral part of the standard of care in several clinical settings, with WB-MRI playing a supportive role. A thorough understanding of the relationship between the treatment landscape and disease volume on conventional imaging, as well as the prognostic significance of prostate-specific antigen (PSA) response, is crucial for determining how higher-accuracy imaging can be effectively incorporated.

In this multidisciplinary collaborative effort by radiologists, nuclear medicine physicians, oncologists, and urologists with expertise in APC, we explore the specific opportunities for high-accuracy imaging in cancer detection and staging, disease characterisation, and aggressiveness assessments, as well as biopsy target selection. The impacts on treatment planning, evaluation of therapeutic response, and theranostics are assessed, and major research questions that need to be addressed in future clinical trials are posed.

## Management landscape of mHSPC

mHSPC can present in men at the time of diagnosis (synchronous) or develop in those who have previously undergone definitive treatment (metachronous) [7]. Generally, patients are classified into low- and high-volume disease states using CHAARTED criteria on conventional imaging or LATITUDE criteria that additionally incorporate the Gleason score [8–10]. Both emphasise the presence of bone and visceral disease as adverse prognostic factors [11, 12], with patients with nodal disease classified as having low volume/risk [4, 5]. High-volume synchronous metastatic disease has the poorest prognosis [7]. Endpoints, including radiographic progression-free survival (rPFS), time to development of castration resistance, and overall survival (OS), are poorer for higher disease volumes [13].

The management of mHSPC primarily stems from clinical trials conducted in synchronous settings. Typically, patients are treated with androgen deprivation therapy (ADT) and androgen receptor pathway inhibitors (ARPI) as a doublet. Chemotherapy-fit patients may benefit from treatment intensification with upfront use of docetaxel (triplet therapy) when the disease burden is high. The therapeutic goal of pelvic radiotherapy (curative or palliative) is also influenced by the volume of metastatic disease, aiming to improve OS for patients with low-volume disease and achieve local tumour control for those with high-volume disease, respectively [14, 15].

## Power of PSA for response assessment and discordance with imaging

Achieving a profound decline in PSA is a critical surrogate marker of treatment efficacy and long-term prognosis in mHSPC. Across multiple randomised controlled trials and real-world data from the International Registry for Men with Advanced Prostate Cancer (IRONMAN), a PSA nadir of < 0.2 ng/mL within the first 6–12 months of systemic therapy has substantial prognostic and predictive value for OS and rPFS [16, 17]. However, correlative imaging studies suggest that biochemical responses alone may not fully capture disease dynamics in men with suboptimal PSA responses in both mHSPC and mCRPC.

### PSMA-PET/CT

A systematic review of 268 mCRPC patients from 10 studies observed discordance between PSA and PSMA-PET/CT responses in approximately one-quarter of cases [18]. Both discordant patterns (imaging progression/biochemical response and biochemical progression/imaging response) were documented across various therapeutic settings and imaging criteria. More recent studies have corroborated these findings [19–21]. For instance, in a prospective study involving 69 mCRPC patients treated with enzalutamide, PSA kinetics and PSMA-PET/CT were only concordant in 48% [20].

### WB-MRI

Similar discordances have been described between WB-MRI and PSA responses, especially in mCRPC. For instance, in a retrospective study evaluating WB-MRI in patients with mHSPC and mCRPC undergoing doublet treatment, all mHSPC patients who achieved a PSA level of ≤ 0.2 ng/mL exhibited no imaging progression [22]. However, non-response or progression was detected in 33.3% of patients who did not achieve a PSA level of ≤ 0.2 ng/mL for mHSPC and in 54.5% of those who did not have a > 50% decrease in PSA levels for mCRPC. WB-MRI depicted disease progression with a suboptimal PSA response had a higher risk of death (hazard ratio, 8.6). Likewise, in a prospective study of mCRPC patients on Olaparib, changes in circulating tumour cell dynamics more strongly correlated with WB-MRI tumour burden than serum PSA [23].

While the clinical implications of discordances between PSA and PSMA-PET/CT or WB-MRI require further investigation, earlier imaging identification of non-responders may assist in avoiding unnecessary toxicity and costs associated with ineffective therapies, allowing for timely transition to alternative treatment strategies when available.

## Biases and the need for clinical trials to assess the clinical impact of high-accuracy imaging

In comparison to conventional imaging, PSMA-PET/CT and WB-MRI enhance both sensitivity and specificity for detecting metastatic disease and assessing suboptimal treatment responses. However, potential biases can create dilemmas in treatment initiations and selections, which include [3, 24–26]:

1. Stage and risk migration. High-accuracy imaging can detect cancer at earlier stages than conventional imaging, leading to earlier treatment exposure for some patients. The reclassification of patients into different groups (e.g., from non-metastatic to metastatic or low-volume to high-volume disease) can inflate group survival rates without an actual improvement of individuals in each group, a phenomenon known as the “Will Rogers effect”.
2. Lead-time bias. High-accuracy imaging can identify the presence of disease or progression earlier than conventional imaging. If earlier therapy initiation for a lower volume of detected disease yields no additional therapeutic benefit, the apparent extended period of survival is spurious, leading to a false impression of improved effectiveness.
3. Length-time bias. High-accuracy imaging can detect slower-growing cancers more effectively than conventional imaging. This can result in apparent longer group survival durations due to the over-detection and over-treatment of lower-risk disease.

To mitigate biases, rigorous methodological approaches are necessary in clinical settings for specific clinical scenarios.

## Opportunities for PSMA-PET/CT and WB-MRI

The opportunities of PSMA-PET/CT and WB-MRI as biomarkers for metastasis detection and staging, disease characterisation, and aggressiveness assessment, as well as biopsy target selection, are highlighted below. While there is no question regarding improved disease detection and staging, it is unclear whether the prognosis of patients whose bone metastases are detected solely through high-accuracy imaging is the same as that of those identified using conventional imaging (i.e., in the absence of tumour engagement with the bone matrix) [25]. We also need to remember that because nodal disease is considered low-volume in the CHAARTED definition [8], the clinical impacts of improved nodal disease detection on systemic therapy and pelvic radiotherapy are uncertain. The clinical benefits of therapy changes due to oligoprogession, shown by high-accuracy imaging, also remain unknown [27]. This highlights the need for prospective clinical studies with surrogate endpoints that demonstrate clinical benefit. In addition, while preliminary studies on cost-effectiveness have demonstrated feasibility, in-depth economic analysis and feasibility of implementing PSMA-PET/CT and WB-MRI with consideration of resource availability and allocation, need further investigation [28–30]. Comparative features of the imaging modalities and central research questions in these clinical domains are highlighted in Tables 1 and 2. Example methodological clinical trials are also highlighted in Table 2.

**Table 1:** Specific features for imaging modalities as biomarkers in advanced prostate cancer clinical domains

Imaging modality/clinical domain	Key features	Biomarker type	Guideline recommendation
**Disease detection and staging**			
PSMA-PET	- Has superior accuracy, sensitivity, and specificity for detecting metastases (lymph nodes, bone, distant). - Redefines “detectable” metastatic disease from anatomical to molecular staging. Identifying microscopic metastatic disease that is not visible on conventional imaging potentially upstages and up-risks patients. - Has high interobserver agreement.	- Diagnostic. - Prognostic (predicting biochemical recurrence-free survival).	- NCCN and EAU guidelines recommend its use for initial staging of intermediate- and high-risk prostate cancer, instead of BS-CT. - ASCO imaging guidelines recommend, as a second step after BS-CT, only when negative or equivocal, or for low-volume disease, if prognosis or management is altered. PSMA-PET or WB-MRI are not recommended when BS-CT scans are definitive regarding the presence of high-volume or widespread M1 disease. This recommendation is generally consistent with AUA/ASTRO guidelines as well.
WB-MRI	- High diagnostic performance for detecting and monitoring metastatic bone disease and soft tissue deposits. Generally, poorer disease detection compared to PSMA-PET, particularly for nodal disease. - Has higher sensitivity and specificity than BS and CT for bone metastases. - In a one-step approach, it can improve T-staging and detect nodal/distant metastases. - Can be used to clarify potential false-positive bone lesions seen on PSMA-PET. May need to be combined with chest CT to screen for lung deposits	- Diagnostic. - Prognostic (providing quantitative insights into tumour biology, e.g., ADC and rFF, related to BCR-free survival).	- EAU highlights the potential for metastatic screening in high-risk prostate cancer. - ASCO imaging guidelines recommend, as a second step after CI, when the result is negative or equivocal, or for low-volume disease, if the prognosis or management is altered. They clearly state that if BS-CT scans are definitive regarding the presence of high-volume or widespread M1 disease, there is no compelling indication for PSMA-PET or WB-MRI. - ESMO recommends staging intermediate- and high-risk disease using conventional MRI or CT, along with a bone scan (BS). - Guidelines acknowledge increased diagnostic performance. Not yet included in the main guidelines due to a lack of Level I-III evidence for widespread adoption.
BS	- Is cost-effective and widely available for total body examination. - Detects areas of active bone formation, particularly around metastases where osteoblastic activity is prominent. - Can detect lesions not visible on CT in some cases.	- Diagnostic (though with high propensity to false-positive uptake).	- Is the current standard for evaluating osseous metastatic disease in intermediate- and high-risk prostate cancer according to EAU guidelines. - It is often used in combination with CT for metastatic screening of intermediate and high-risk patients. - ASCO imaging recommends its use as a conventional imaging option for all patients with APC. - It is recommended by EAU for metastatic screening if PSMA-PET/CT is not available.
CT	- Requires substantial cortical destruction/remodelling for visibility. - Is useful for assessing questionable bone scan findings by demonstrating benign conditions (e.g., trauma, degenerative changes) that result in false-positive appearances on bone scan. - Is mainly used for evaluating chest or abdominopelvic lymph-node metastasis. - Low-dose whole-body CT as part of PET evaluations can help classify patients and potentially eliminate the need for multiple imaging sessions and high-cost scans for high-burden disease.	- Diagnostic (though with limitations for bone metastases and micrometastases, as cortical destruction is needed for visibility).	- Is recommended by EAU for cross-sectional abdominopelvic imaging for metastatic screening. - Is often used in combination with BS. - ASCO recommends CT as a conventional imaging option for all patients with APC.
**Disease characterisation**			
PSMA-PET	- Provides crucial functional and metabolic information about tumours. - Higher SUVmax correlates with aggressive disease and less favourable biochemical recurrence-free survival. - Can indicate cancer’s aggressiveness and likelihood to spread. - Helps identify active bone metastases suitable for CT-guided biopsy	- Prognostic. - Diagnostic (for identifying active lesions and guiding biopsy).	- Is not specified for aggressiveness assessment.
WB-MRI	- Offers quantitative insights into tumour biology, especially with ADC and rFF mapping. - Has high specificity for the presence of active bone disease using multiparametric features like hyperintensity on high b-value DWI, low ADC and rFF values. - Helps identify active bone metastases suitable for CT-guided biopsy	- Prognostic. - Diagnostic (for identifying active lesions and guiding biopsy).	- Is not specified for aggressiveness assessment.
BS	- Assesses reactive osteoblastic uptake, but does not directly evaluate malignant bone disease or pure lytic metastases.	- Limited for aggressiveness assessment.	- Is not specified for aggressiveness assessment.
CT	- Has limitations for direct evaluation of malignant bone disease without a soft tissue component. - CT-guided biopsy is used to obtain tissue for next-generation genomic sequencing and molecular analysis. - Multiple CT scan features can be used to define aggressive prostate cancer variants.	- Diagnostic (limited for aggressiveness assessment).	- Is not specified for aggressiveness assessment.
**Impacting treatment management**			
PSMA-PET	- Enables personalised treatment planning by providing precise information about the tumour location and extent. - Is instrumental in guiding the selection of patients for PSMA-RLT. Low or absent PSMA uptake in known metastatic sites suggests that the patient may not benefit from PSMA-targeted RLT. - Refines radiation treatments by improving target identification and dose management, and allowing for dose escalation to disease not visible with conventional imaging. - Informs the development and updating of pelvic lymph node contouring guidelines. - Can guide highly focused therapies, such as stereotactic body radiation therapy (SBRT), to specific regions (metastasis-directed therapy).	- Diagnostic (for guiding therapy choice). - Prognostic (higher volume of PSMA-expressing disease confers worse survival) - Predictive (higher PSMA SUVmean predictive for RLT response and patient selection).	- ASCO advises considering the imaging modality to guide treatment or change clinical treatment decisions. - PSMA PET is used for patient selection for PSMA-Radioligand Therapy (PSMA-RLT).
WB-MRI	- Enables guiding MDT in oligometastatic PCa. - Provides detailed anatomical and functional information, allowing for a more personalised approach to prostate cancer management. - Can help confirm bone metastatic disease by excluding PSMA false-positive lesions.	- Diagnostic (for guiding therapy). - Predictive (for MDT).	- Is not specified for treatment planning.
BS	- Primary tumour radiotherapy treatment decisions are often based on conventional imaging findings, which include BS. - Disease volume on BS-CT is used to inform the systemic anticancer therapy approach.	- Prognostic. - Predictive.	- NCCN and EAU guidelines recommend conventional imaging with BS-CT to guide prostate radiotherapy in addition to systemic therapy.
CT	- Primary tumour radiotherapy treatment decisions are often based on conventional imaging findings, which include CT. - Disease volume on BS-CT is used to inform the systemic anticancer therapy approach. - Is often used as part of PSMA PET/CT for anatomical correlation. Contrast-enhanced CT or MRI may be done before RLT to detect PSMA-negative disease and consider its extent.	- Diagnostic (provides anatomical context for theranostics). - Prognostic - Predictive.	- NCCN and EAU guidelines recommend conventional imaging with BS-CT to guide prostate radiotherapy in addition to systemic therapy.
**Disease monitoring and response assessment**			
PSMA-PET	- Can determine if the disease is regressing, stable, or progressing, allowing timely identification of treatment failure. - PPP (for limited disease/mHSPC) and RECIP (for advanced disease/mCRPC) were developed to standardise the interpretation of responses. PROMISEv2 framework proposes standardised parameters for longitudinal reporting of PSMA-PET using PPP, RECIP and tumour volume assessments - Can indicate treatment success or emerging resistant disease before clinical or biochemical failure becomes obvious.	- Response. - Prognostic.	- ASCO guideline notes that advanced imaging, such as PSMA-PET or WB-MRI, may play a role if performed at baseline, facilitating comparison of subsequent imaging findings and assessing the extent of progression. - PSMA PET/CT: joint EANM guideline/SNMMI procedure standard suggests the potential for response monitoring, including PSMA-targeted RLT.
WB-MRI	- Objective measures, such as relative T1-weighted MRI signal intensity and rFF, correlate with survival and biochemical progression. - Allows for the objective measurement of nodal, visceral, and skeletal bone metastases, as well as their response to therapy. - Multiparametric assessments encompassing diffusion signal intensity, ADC values and rFF is needed for successful response interpretations.	- Response. - Prognostic (an increase in tumour fat is a powerful prognostic factor for a longer response. - MET-RADS-P response assessment categories correlate with the risk of death.	- ASCO guideline notes that advanced imaging, such as PSMA-PET or WB-MRI, may play a role if performed at baseline, facilitating comparison of subsequent imaging findings and assessing the extent of progression.
BS	- Historically used to assess progression only, but has significant limitations, including low specificity, inability to evaluate soft tissue or pure lytic lesions, and the flare phenomenon (mimicking progression due to healing). - PCWG3 criteria are reproducible, easy to apply, and account for the flare response.	- Response (limited utility for bones only). - Prognostic.	- PCWG3 criteria for determining progression require identifying at least two new lesions on the first assessment following a baseline scan and at least two further new lesions on a subsequent confirmatory scan.
CT	- Requires substantial changes in bone density to distinguish between osteoblastic healing and osteoblastic progression after treatment.	- Response (limited utility for bone). - Prognostic.	- APCCC and ASCO imaging guidelines suggest that conventional imaging should be used at PSA progression in mHSPC. Regular imaging is advised for mCRPC. - For soft tissue (nodal and visceral) disease, PCWG3 criteria incorporate RECIST 1.1 criteria for assessing response. - There are no CT scan-specific criteria for assessing bone treatment response. - According to RECIST 1.1, bone lesions are considered non-measurable disease.

*ADC* apparent diffusion coefficient, *APCCC* Advanced Prostate Cancer Consensus Conference, *ASCO* American Society of Clinical Oncology, *ASTRO* American Society for Radiation Oncology, *AUA* American Urology Association, *BCR* biochemical recurrence, *BS* bone scan, *CI* conventional imaging, *CT* computed tomography, *DWI* diffusion-weighted imaging, *EAU* European Association of Urology, *ESMO* European Society of Medical Oncology, *MET-RADS-P* METastasis Reporting and Data System for Prostate Cancer, *NCCN* National Comprehensive Cancer Network, *PCa* prostate cancer, *PCWG3* Prostate Cancer Clinical Trials Working Group 3, *PET* positron emission tomography, *PPP*  PSMA PET progression, *PROMISE* prostate cancer molecular imaging standardised evaluation, *PSMA* prostate-specific membrane antigen, *RECIST* response evaluation criteria in solid tumours, *rFF* relative fat fraction, *RLT* radioligand therapy, *RECIP* response evaluation criteria in PSMA PET/CT, *SUVmax* maximum standardised uptake value, *WB-MRI* whole-body magnetic resonance imaging

**Table 2:** High-accuracy imaging, key research questions, and clinical trials in advanced prostate cancer

Major research questions	Clinical trials
**Disease detection and staging**	
- How does disease volume depicted on PSMA-PET or WB-MRI relate to disease volume seen on conventional imaging? - How can the disease burden on PSMA-PET or WB-MRI be optimally applied to clinical practice or trials? - How can false positives on PSMA-PET be more reliably differentiated from actual metastatic disease to avoid over-interpretation and unnecessary interventions? - What are the optimal imaging protocols and interpretation criteria for PSMA-PET and WB-MRI to maximise diagnostic accuracy and minimise inter-reader variability for nodal and distant metastases? - What is the long-term impact of PSMA-PET guided initial staging for metastatic disease on patient outcomes, including metastasis-free survival and quality of life? - How can the low detection sensitivity for identifying small lymph node metastases be incorporated into multivariable risk calculators for specific patient subgroups in de-escalation strategies aimed at avoiding pelvic lymph node dissection? - What are the optimal complementary roles of PSMA-PET and WB-MRI in initial staging, particularly for detecting pelvic nodal and distant metastases? - How can the suboptimal capability of WB-MRI for assessing lung metastases be improved? - How can AI improve image quality, lesion detection, segmentation, and lesion tracking in PSMA-PET/CT and WB-MRI? - Can nanoparticle-MRI using ultrasmall superparamagnetic iron oxide contrast agent be used to improve nodal staging of WB-MRI?	- The AVIDITY study will assess the clinical utility of PSMA-PET/CT in staging patients with newly diagnosed high-risk PCa compared to conventional imaging [99]. The primary endpoint, MFS after three years, aims to demonstrate whether PSMA-PET-guided staging leads to improved clinical outcomes.
**Disease characterisation**	
- How can quantitative imaging biomarkers from PSMA-PET and WB-MRI be better integrated with molecular biomarkers to characterise tumour aggressiveness and heterogeneity in patients with mHSPC more accurately? - How can combined imaging and molecular insights predict treatment response and long-term outcomes more effectively? - What are the optimal thresholds or imaging patterns on PSMA-PET and WB-MRI that reliably differentiate patients likely to have a more indolent from an aggressive disease course?	- The STAR-TRAP trial will evaluate the role of PSMA-PET and WB-MRI-directed MDT in men with oligometastatic disease at biochemical failure or after initiation of systemic therapy for polymetastatic disease, provided they have a good but incomplete response [100]. This research aims to determine the value of PSMA-PET and WB-MRI for radiotherapy consolidation and the treatment of early disease recurrence.
**Impacting treatment management**	
- How effectively does PSMA-PET and WB-MRI-guided treatment intensification in mHSPC lead to improved long-term patient outcomes compared to conventional imaging-guided approaches? - How can advanced imaging be integrated into adaptive radiotherapy planning to account for tumour changes in nodal and metastatic sites during treatment, and what is the clinical benefit of such adaptive strategies? - How can PSMA-PET serve as a predictive biomarker for the efficacy of PSMA-targeted RLT, and what are the optimal patient selection criteria based on imaging characteristics to maximise therapeutic benefits? - What is the role of PSMA-PET in guiding systemic therapy selection and sequencing for identifying patients who might benefit from PSMA-targeted therapies? - What imaging response criteria for PSMA-PET predict clinical outcomes in patients undergoing RLT? - How does the greater sensitivity of PSMA-PET, when used to monitor RLT, improve long-term patient outcomes compared to using conventional imaging alone? - In what ways can brief administration of standard androgen receptor antagonists and the associated PSMA “flare” phenomenon be utilised to augment PSMA expression, thereby improving the efficacy of PSMA-targeted radioligand therapy? - How can WB-MRI play an additional role, especially when there is disagreement between PSMA-PET and PSA, or when evaluating for the potential development of non-PSMA-avid disease?	- The PATRON (NCT04557501) trial compares conventional imaging with PSMA-PET-guided treatment intensification (radiotherapy or surgery) in patients with high-risk, untreated PCa or biochemically recurrent PCa, aiming to provide Level 1 evidence on whether PSMA-PET-guided management improves 5-year failure-free survival [101]. - The DECREASE trial will compare darolutamide only versus darolutamide + local consolidation radiotherapy to PSMA+ sites in patients with conventional imaging-defined non-metastatic (M0) CRPC. - The NCI-2023-00612 (NCT05683964) Phase 2 trial is investigating whether a short course of standard ADT induces a PSMA “flare” phenomenon, potentially providing a new approach to enhance PSMA expression for more effective PSMA-targeted RLT [102]. It also examines the effectiveness of PSMA PET/CT-guided para-aortic radiation therapy combined with short-term androgen suppression therapy for oligorecurrent disease. - Several randomised phase 2/3 trials are exploring whether combining various types of systemic therapies (ADT +/− ARPI) with MDT to oligometastatic disease or progression identified on PSMA-PET. Examples include the ADOPT trial (MDT + ADT versus ADT) [103], the PROMETHEAN trial (MDT + ADT versus MDT) [104], the VA STARPORT trial (MDT + ADT +/− ARPI versus ADT +/− ARPI) [105], and the PERSIAN trial (MDT + ADT + ARPI versus ADT + ARPI) [106]. These trials will assess several outcomes such as OS, rPFS, MFS, and QoL. - Phase 3 trials, such as UpFrontPSMA [107] and PSMAddition [108], are examining its use in patients with PSMA-positive mHSPC, comparing outcomes like rPFS and OS between those receiving PSMA-targeted RLT plus standard of care and those receiving standard of care alone. - The Phase 3 STAMPEDE2 trial (NCT06320067, ISRCTN66357938) includes an embedded imaging sub-study that will evaluate treatment response after PSMA-targeted RLT, using paired PSMA-PET and WB-MRI, with endpoints of rPFS and OS.
**Disease monitoring and response assessment**	
- How do you distinguish between actual disease progression and therapy-induced “flare phenomena” on PSMA-PET scans, given that systemic therapies may alter PSMA expression? - What are the optimal response criteria for PSMA-PET and WB-MRI that reliably predict clinical outcomes in metastatic prostate cancer? What level of change in PSMA or WB-MRI biomarkers best predicts clinical benefit? - What are the optimal timings for PSMA-PET and WB-MRI in metastatic treatment response settings versus PSA, and do these differ between mHSPC and mCRPC? - Does the earlier detection of biochemical recurrence or treatment failure using PSMA-PET or WB-MRI and treatment switching lead to improved long-term outcomes? - What are the roles of serial PSMA-PET and WB-MRI assessments for monitoring suboptimal response, disease progression, and for earlier treatment switching? Do earlier therapy changes lead to clinically meaningful patient outcomes?	- The ADRRAD trial investigates the correlation between WB-MRI-based T1-weighted signal intensity changes and clinical outcomes, such as survival [109]. - The PSMAtrack trial evaluates the efficacy of serial PSMA-PET/CT scans in monitoring treatment responses and identifying correlations between residual PSMA-avid disease and biomarkers, such as PSA [110]. - The PEACE-6 study examines PSMA-PET-guided treatments, comparing standard continuous androgen blockade with intermittent blockade in patients who are deep PSA responders without employing PSMA-PET in assessments [111]. However, it also explores the intensification of therapy with PSMA-targeted RLT for patients who are poor responders to standard therapies.

*ADC* apparent diffusion coefficient, *ADT* androgen deprivation therapy, *ARPI* androgen receptor pathway inhibitor, *BCR* biochemical recurrence, *CRPC* castration-resistant prostate cancer, *FDG* fluorodeoxyglucose, *MDT* metastasis-directed therapy, *MET-RADS-P* METastasis Reporting and Data System for Prostate Cancer, *MFS* metastasis-free survival, *mHSPC* metastatic hormone-sensitive prostate cancer, *OS* overall survival, *PCa* prostate cancer, *PET* positron emission tomography, *PPP*  PSMA PET progression, *PSA* prostate-specific antigen, *PSMA* prostate-specific membrane antigen,* PSMA-TV*  PSMA-derived tumour volume, *QoL* quality of life, *rFF* relative fat fraction, *RLT* radioligand therapy, *RECIP* Response Evaluation Criteria in PSMA PET/CT, *rPFS* radiographic progression-free survival, *SUVmax* maximum standardised uptake value, *TL-PSMA* total lesion PSMA, *WB-MRI* whole-body magnetic resonance imaging

### Disease detection and staging

#### PSMA-PET/CT

**Fig. 1:**
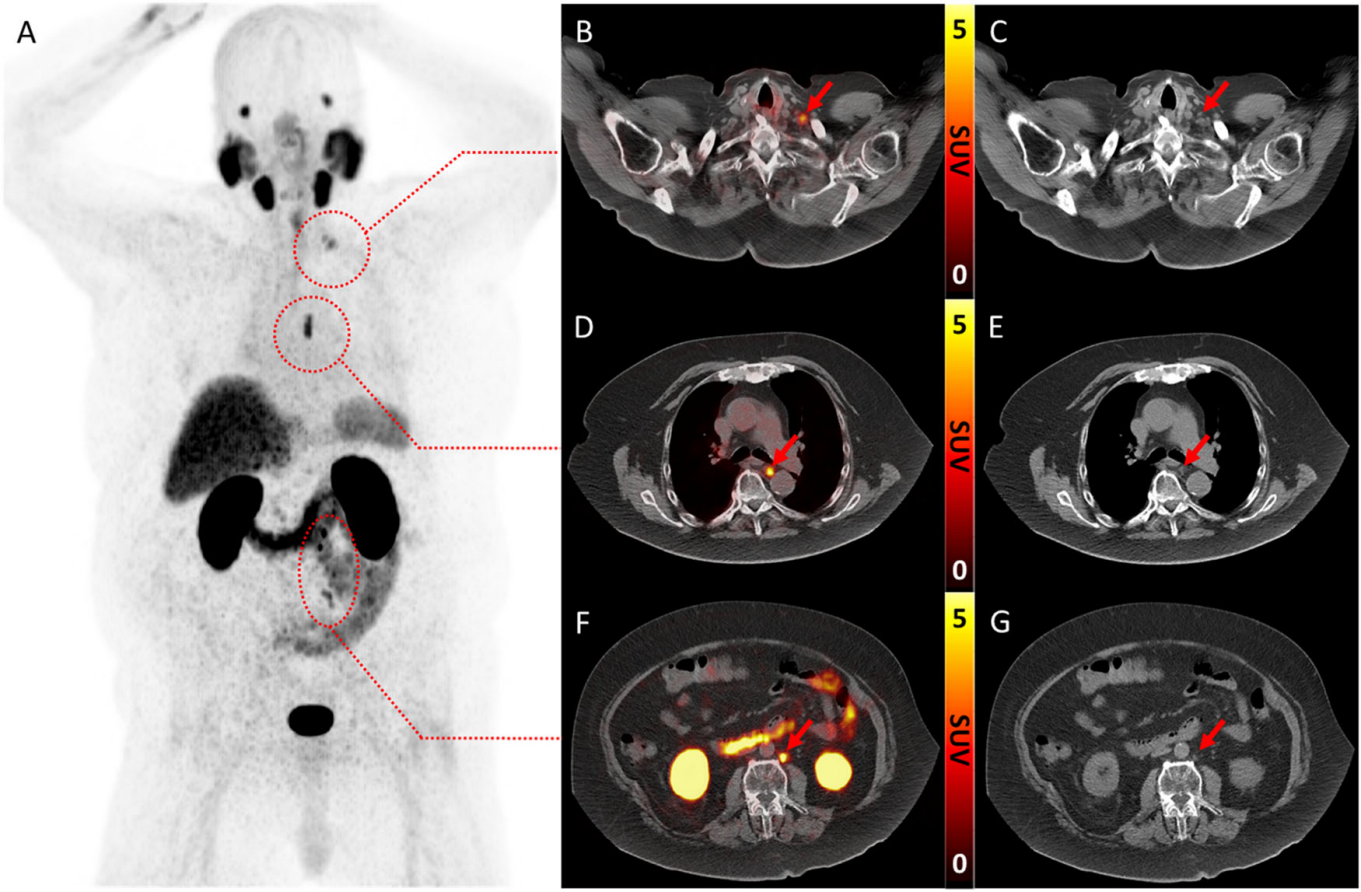
Improved sensitivity of PSMA-PET/CT leading to *upstaging*. An 81-year-old man with Grade Group 4 prostate cancer previously treated with radiotherapy and now with biochemical recurrence (PSA 4.9 ng/mL). **A** Maximum-intensity projection (inverted grey scale) image of PSMA-PET shows PSMA-expressing retroperitoneal, mediastinal, and left supraclavicular lymph nodes (arrows). On axial images, fused PET/CT (middle) and CT (right) images show that these nodes are not enlarged (**B**, **C** left supraclavicular; **D**, **E** mediastinal; **F**, **G** left paraaortic), demonstrating *upstaging* from M0 (on CT) to M1a (on PSMA-PET/CT)

PSMA-PET/CT has greater accuracy in detecting metastatic disease than BS-CT. In patients with high-risk PCa, the ProPSMA trial demonstrated that PSMA-PET/CT had higher sensitivity (85% versus 38%) and specificity (98% versus 91%) for detecting pelvic nodal and distant metastases [31]. The improved accuracy of PSMA-PET/CT not only leads to upstaging/up-risking (due to higher sensitivity) but also to downstaging/down-risking (due to higher specificity) (Fig. 1). For example, 57% of positive bone scans were false positives when compared with PSMA-PET at initial staging [32]. Unterrainer et al [33] evaluated 67 patients with mHSPC who had at least M1a disease on conventional imaging. They noted that 24% of patients with M1 disease had only local (N0) or pelvis-confined (N1) disease on PSMA-PET/CT, with risk assessment changes occurring in 42% (24% down-risked and 18% up-risked).

#### WB-MRI

Lecouvet et al [34] demonstrated in patients with high-risk PCa that WB-MRI has higher sensitivity than BS-CT (98–100% versus 86%) for detecting bone metastases and similar sensitivity to CT (77–82% for both) for detecting metastatic nodes. A comparative study between PSMA-PET and WB-MRI showed that while the capability for detecting distant metastasis was similar, WB-MRI had a slightly inferior ability to detect nodal metastases [35]. WB-MRI also alters the risk burden of men with mHSPC, primarily related to the higher detection of bone-only metastases. In more than 200 age-matched patients, Hassan et al [36] noted that WB-MRI risk classification was more effective than BS-CT for predicting overall survival in men with mHSPC. The tumour burden depicted on WB-MRI was also found to be prognostic in mCRPC [37, 38].

### Disease characterisation

#### Identifying biopsy targets for molecular analysis

Rebiopsy and genomic analysis are recommended for patients exhibiting intrinsic or acquired resistance to guide treatment and inform potential trial involvement. For patients with metastatic PCa, bone biopsies are often the only source for molecular analysis of actionable mutations. Successful tissue sampling can aid in identifying mutations that inform personalised treatments [39].

#### PSMA-PET/CT

Several studies have demonstrated that PSMA-PET/CT-informed bone biopsy results in high success rates for molecular analysis, ranging from 66% to 70% [40, 41]. High maximum standardised uptake values (SUVmax) at PSMA-PET and low Hounsfield Units (HUs) at CT are strong predictors of success. In a study of 69 patients who underwent PSMA-PET/CT, samples suitable for whole-genome sequencing had a median SUVmax of 20.9 and a HU of 786 [41]. Donners et al [42] also noted that HU affected histological yields, with a 610 HU threshold having a positive predictive value of 89% for tumour-positive biopsies and a 370 HU threshold for successful next-generation sequencing.

#### WB-MRI

Similarly, a prospective study of 20 patients using multiparametric WB-MRI assessments found that 85% of samples were positive for bone metastasis; 72% were suitable for genomic sequencing [43]. Using biopsy yields in 43 patients evaluated on WB-MRI, the combination of hyperintensity on high b-value diffusion-weighted images (DWI), apparent diffusion coefficient (ADC) values < 1100 μm^2^/s, and a relative fat fraction (rFF) of < 20% based on the T1-weighted Dixon technique had a PPV of 93% [44]. Another report on 10 patients with mCRPC showed that combining multiparametric PSMA-PET/CT and WB-DWI had success rates of 90% for positive biopsy and 80% for successful molecular analysis [45].

#### Characterising aggressive disease variants

**Fig. 2:**
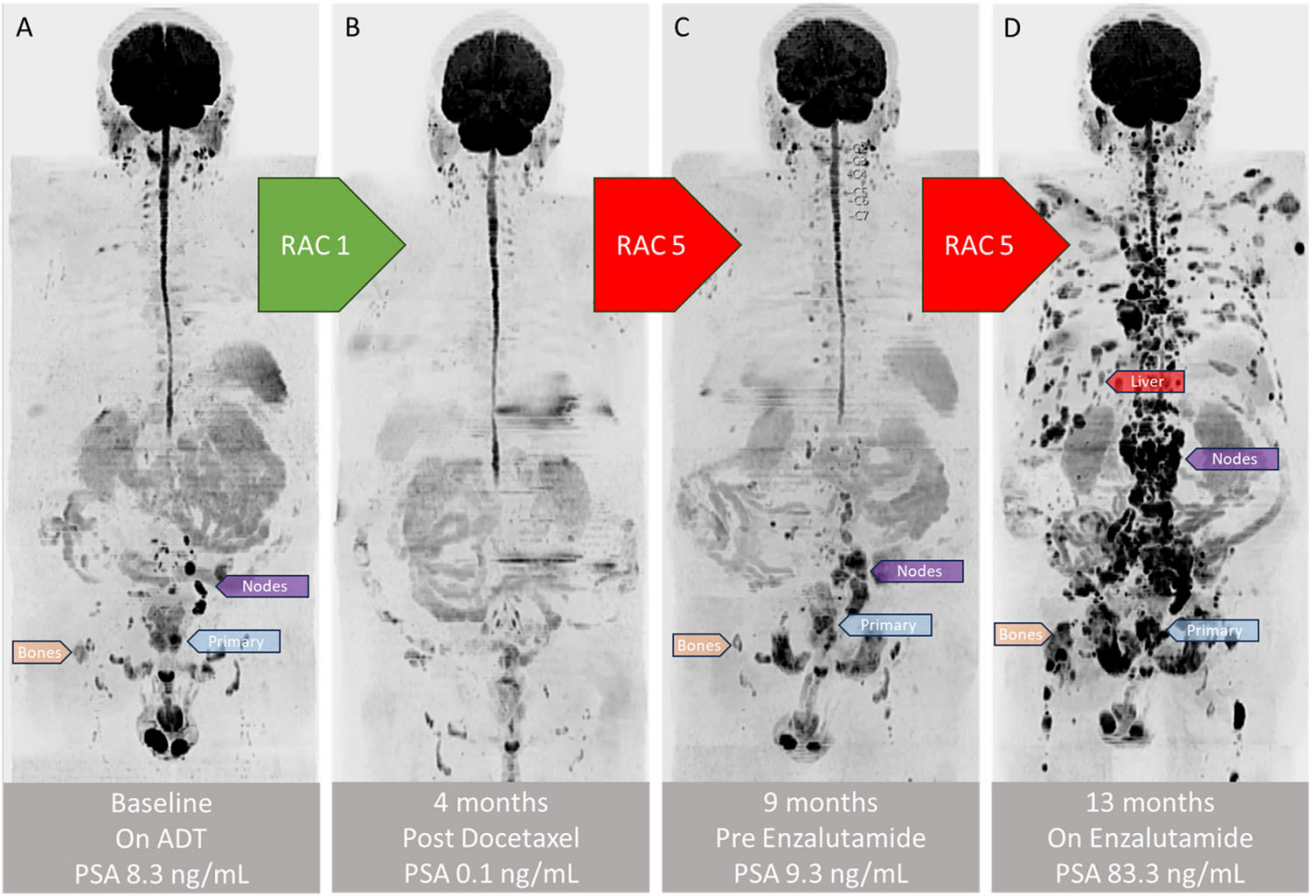
WB-MRI (inverted b-900 maximum-intensity projection images) demonstrating the emergence of an aggressive clinical phenotype after targeted therapy. **A** A 65-year-old man with synchronous polymetastatic locally advanced prostate cancer (Grade Group 5 with PSA 8.3 ng/mL, T3bN1M1aM1b). The primary prostate tumour, nodal metastases, and bone metastases are annotated with blue, purple, and yellow arrows, respectively. **B**, **C** After an initial response to androgen deprivation therapy and docetaxel (Response Assessment Category (RAC) 1, PSA 0.1 ng/mL), progression occurred 9 months after initiation of systemic therapy (RAC 5, PSA 9.3 ng/mL). **D** Four months after subsequent treatment with an androgen receptor pathway inhibitor, rapid progression was noted (RAC 5, PSA 83.3 ng/mL) with widespread disease involving nodes, bones, and the liver (red arrow). Note: Response Assessment Categories (RACs) on WB-MRI grade metastatic disease: RAC 1-2 indicates the extent of likely response, RAC 3 is stable disease (active or inactive), and RAC 4-5 indicates the extent of likely progression. These are assessed using morphology and DWI (including ADC values) for bone and soft tissue, compared against a baseline or nadir scan, consistent with RECIST v1.1 principles

While ARPIs are the backbone of mHSPC treatment, 20–25% of patients do not experience durable responses beyond 2 years [46–48]. Aggressive histologic and clinical phenotypes can emerge, which are less dependent on androgen receptor signalling, including neuroendocrine, small cell, and aggressive variant adenocarcinomas (Fig. 2). Multiple molecular events (e.g., PTEN deletion, RB1 loss, and p53 deficiency; intrinsic or as a result of effective androgen receptor blockade) can contribute to lineage plasticity, resulting in the appearance of treatment-emergent aggressive variants [49]. Conventional imaging features can suggest the presence of aggressive variants, such as the presence of bulky metastatic disease, predominant lytic bone metastases, and very low PSA levels when associated with a high tumour volume [50]. The role of WB-MRI or PSMA-PET/CT for identifying and assessing the therapy response of aggressive disease variants is not well established. WB-MRI can be effective because it evaluates tissue cellularity and bone marrow replacement. On the other hand, early studies indicate that aggressive variants may downregulate PSMA expression [51] and upregulate glycolytic metabolic activity, enabling [18 F]-fluorodeoxyglucose (FDG)-PET to serve as a prognostic biomarker [52]. Furthermore, an integrated assessment using multiple PET radioligands (e.g., upfront FDG-/PSMA-PET followed by DOTATATE-PET) may provide prognostic information to assist in decision-making [53].

### Impacting treatment management

The higher accuracy of PSMA-PET/CT is refining radiotherapy (RT) planning at initial staging, for salvage and metastasis-directed radiotherapy [54].

#### Metastasis-directed therapy for oligometastatic disease

The ability to accurately identify small lesions can guide highly focused therapies, such as stereotactic body radiation therapy, thereby minimising collateral damage to healthy tissues. Oligometastatic disease represents an intermediate state in which targeted local therapy can be beneficial [55]. Both PSMA-PET/CT and WB-MRI are used for patient selection and to guide metastasis-directed treatments (MDT) in patients who typically have five or fewer metastatic sites of disease [55]. MDT has demonstrated favourable disease-free survival and OS, particularly for patients with low-volume bone disease in the metachronous mHSPC setting, especially when informed by PSMA-PET/CT [56–58].

The PEACE V-STORM study showed that the MDT approach may not be equally applicable to oligo-metachronous PET-detected nodal recurrences. In this Phase 2 randomised study comparing MDT and regional radiotherapy, metastasis-free survival was better with regional radiotherapy, presumably because microscopic metastatic disease is not seen [59]. This result is consistent with the findings of the POP-RT study, which also noted that pelvic node radiotherapy cannot be omitted based solely on negative PSMA-PET/CT results, given the low node detection sensitivity of 58% (95% confidence interval, 50–66%) [60, 61]. The role of MDT for oligoresidual disease and oligoprogressive disease is under investigation (Table 2).

#### Optimising radiotherapy fields and surgical approaches

MRI-guided focal boost radiotherapy involves delivering a higher dose of radiation to specific areas of the prostate, identified as tumours on MRI. This treatment improves treatment outcomes, such as biochemical disease-free survival, and potentially reduces the risk of recurrence, without significantly increasing side effects [62].

PSMA-PET/CT can play a significant role in refining radiotherapy by enhancing target identification and dose escalation to the disease that may not have been visible with conventional imaging [63]. The long-term treatment outcomes of PSMA-PET/CT-guided radiotherapy plans are currently under investigation (Table 2). Although WB-MRI using diffusion-weighted sequences is excellent for depicting the location of all lymph nodes, its use for radiotherapy planning has not been explored due to its poor sensitivity for detecting involved nodes.

PSMA-radioguided surgery may also assist surgeons in identifying involved lymph nodes in patients undergoing extended pelvic lymph node dissection and for sentinel lymph node sampling [64, 65]. However, further follow-up data are needed to assess the impact of PSMA-radioguided surgery on long-term oncological outcomes.

#### PSMA-theranostics

PSMA-targeted radioligand therapy (RLT) specifically targets PSMA expressed on the surface of malignant cells. PSMA-PET imaging plays a pivotal role in identifying suitable candidates for treatment by enabling the visualisation of PSMA expression in disease sites. The prognostic and predictive value of baseline PSMA-PET imaging with PSMA-targeted RLT is being established [66–68]. Patients with metastases demonstrating a higher degree of PSMA expression are considered to have a greater likelihood of benefiting from PSMA-targeted RLT [69]. The predictive ability of PSMA uptake was not shown for the combined use of ARPI and RLT [70]. The European Association of Nuclear Medicine (EANM) and Society of Nuclear Medicine and Molecular Imaging (SNMMI) have published procedure guidelines for the use of PSMA-targeted RLT, encompassing eligibility criteria, patient selection, the treatment process, and follow-up [71]. While PSMA-PET is valuable in its own right, evidence suggests that combining FDG-PET with PSMA-PET, although not mandatory, may enhance patient selection and better predict treatment outcomes compared to using PSMA-PET alone by excluding patients who have discordant PSMA-negative and FDG-positive disease [71, 72]. Readers should note that PSMA-targeted RLT is currently approved based on PSMA-PET positivity alone [73–76].

### Disease monitoring and response assessments

Evaluating the treatment response of PCa patients with metastatic bone disease is challenging. BS-CT report on the interactions of marrow disease with mineralised bone, primarily through osteolytic and osteosclerotic mechanisms, which may not reliably indicate treatment efficacy [77]. Apparent worsening of BS-CT (“flare reactions”) is frequently observed when patients respond clinically [78]. On the contrary, clinical deterioration not observed with BS-CT is also common [79]. Furthermore, BS/CT progression without PSA progression has also been repeatedly demonstrated in mHSPC with ADT alone and combined with enzalutamide and apalutamide treatments [46, 47, 80]. These findings support the NCCN v1.2025 guideline for periodic imaging to monitor treatment of mHSPC, which currently does not endorse the use of PET imaging in this context. However, both PSMA-PET/CT and WB-MRI can overcome these limitations because the tumour response within the marrow space is directly depicted [23, 81].

#### PSMA-PET

PSMA-PET response assessment criteria, such as PSMA PET Progression (PPP) and Response Evaluation Criteria in PSMA PET/CT (RECIP), were developed to standardise interpretations of responses to RLT [82, 83]. Progression using the PSMA PET-specific criteria after PSMA-targeted RLT has been significantly associated with shorter overall survival (OS) [84, 85]. The RECIP framework employed an evidence-based approach that considers changes in total tumour volume (TTV). RECIP 1.0 categorises scans into response assessment categories based on changes in PSMA-positive TTV and the appearance of new lesions. RECIP 1.0 has prognostic value for OS and exhibits excellent interreader agreement for both visual and quantitative assessments [86]. It was initially developed for late-stage mCRPC but has been successfully used in mHSPC for monitoring ARPI use [87]. The Prostate Cancer molecular imaging standardised evaluation (PROMISE) v2 framework proposes standardised parameters for longitudinal reporting of PSMA-PET using PPP, RECIP, and tumour volume assessments [88].

Tumour response assessments after ADT or ARPI may pose a specific challenge for PSMA-PET. Suppression of androgen receptor signalling can increase PSMA expression (“flare phenomenon”) even as the tumour responds, especially in mCPRC [89]. On the other hand, this can also cause a decrease in PSMA expression [90]. Such modulations in PSMA expression can make it difficult to interpret changes in PSMA uptake reliably, and further studies are needed to clarify the role of PSMA-PET in this context. There is emerging data on PSMA-PET for monitoring chemotherapy response [91]. The optimal timing for end-of-treatment versus interim imaging remains under investigation, and clear guidelines for timing have yet to be established. Data on PSMA-PET as a response biomarker for other systemic therapies (e.g., PARP inhibitors, Radium-223) are limited, with early studies suggesting a potential role [92], but further investigations are required.

#### WB-MRI

Multiparametric tumour response assessments using WB-MRI potentially offer more precise differentiation of bone metastasis response with a lesser susceptibility to flare responses compared to those using PSMA-PET. Multiple WB-MRI studies have reported that changes in tumour volume, ADC values, and rFF% are associated with clinical response [44, 45]. These parameters are incorporated into the METastasis Reporting and Data System for Prostate Cancer (MET-RADS-P) [93]. Garcia-Ruiz et al [94] evaluated quantitative WB-MRI biomarkers for their ability to predict bone disease progression in patients with metastatic PCa treated mainly with ARPI. An increase in tumour fat was the most powerful prognostic factor for a more extended response. Interestingly, changes in ADC values were not predictors of survival benefits. In addition, response assessment categories (RACs) - which include multiparametric assessments of morphological findings, ADC, and rFF% were predictive of treatment benefits in a secondary analysis [22].

A prospective study of 109 patients with mHSPC receiving enzalutamide reported a high bone response rate, with 80% achieving complete/partial responses (RAC 1–2) at 6-12 months [95]. PSA responses were consistent with MRI in 78.5% of cases (Cohen’s *k* of 0.324). Critically, lower RAC scores correlated with a lower risk of death, with a hazard ratio of 0.15. The interrater agreement for RAC scoring for bone disease is substantial to excellent [96].

RECIP and MET-RADS-P represent distinct frameworks for assessing treatment response, distinguished by their underlying principles and practical requirements. RECIP quantitatively assesses changes in TTV against a baseline scan and is sensitive for tracking only PSMA-positive disease; however, as noted above, its reliance on measuring the therapeutic target itself makes it vulnerable to therapy-induced biomarker modulation and potentially blind to PSMA-negative resistance. In contrast, the therapy-agnostic MET-RADS-P framework assesses the downstream biological effects of treatments, such as changes in tumour cellularity (ADC) and marrow composition, comparing against either baseline or nadir scans to robustly detect actual cell death and repair mechanisms regardless of the treatment mechanism. This fundamental difference extends to their implementation: RECIP’s volumetric analysis may need specialist software for clinical trials (although visual assessments may be equally effective for clinical practice [86]. MET-RADS-P uses standard radiological tools, making it highly accessible and practical for routine clinical practice. While both assessment methods are superior to conventional imaging, there is a lack of comparative analysis between PSMA-PET and WB-MRI [84, 97].

## Conclusion

The increasing availability of PSMA-PET/CT and WB-MRI has undeniably transformed the detection and characterisation of APC. While these tools offer detailed biomarker information, their potential to fundamentally alter the disease course and improve long-term patient outcomes via treatment adaptations remains unproven. While their strong rule-in ability for identifying new disease sites makes therapy escalations generally safer, radiologists and clinicians must exercise caution with therapy de-escalations, given their moderate rule-out capability [98]. The availability of treatments for micrometastatic disease detected by PSMA-PET/CT and WB-MRI may not inherently translate into an altered clinical risk-to-benefit ratio for all patients, underscoring the urgent need for robust studies that integrate imaging with therapeutic interventions. Furthermore, the common discordance between clinical assessments, PSA measurements, and BS-CT in accurately depicting bone disease progression highlights a critical need for vigilance and regular, protocol-based response assessments. Both PSMA-PET/CT and WB-MRI offer a more accurate reflection of treatment-induced changes, encompassing both response and progression, with their respective response criteria currently undergoing validation and demonstrating prognostic value (Table 3). Prospective clinical trials will be required to evaluate whether higher accuracy imaging can genuinely improve clinically relevant endpoints for patients through precise treatment adaptations.

**Table 3:** ESUR statements on the use of PSMA-PET and WB-MRI in advanced prostate cancer

Clinical Domain	Statements
Disease detection and staging	**Initial staging:** - PSMA-PET preferred and recommended for newly diagnosed high-risk and locally advanced prostate cancer to detect metastatic disease, often as a front-line tool, with conventional imaging not always a prerequisite. - All-in-one prostate and WB-MRI enhances staging accuracy and risk stratification compared to conventional imaging.
	**Detection of locoregional and distant metastases:** - PSMA-PET consistently demonstrates superior sensitivity and specificity compared to conventional imaging for detecting both nodal and distant metastatic lesions. PSMA-PET outperforms WB-MRI in distant staging accuracy. - WB-MRI is highly effective for detecting metastatic disease, particularly bone metastases, often outperforming bone scintigraphy and identifying higher rates of bone-only, high-volume, high-risk, and *de novo* metastatic disease compared to conventional imaging.
Disease characterisation	**Enhanced tumour characterisation:** - PSMA-PET provides detailed tumour assessments of metastatic sites, with various PET parameters correlating with disease aggressiveness and outcomes such as progression-free and overall survival. - WB-MRI provides multiparametric quantitative insights with high specificity for the presence of active disease and genomic characterisation.
	**Supporting advanced radiotherapeutic approaches:** - The diagnostic precision afforded by both PSMA-PET and WB-MRI enables advanced therapeutic approaches, including metastasis-directed therapy for patients with oligometastatic disease.
Impacting treatment management	**Metastasis-directed therapy:** - The ability to accurately identify small, previously undetected lesions on conventional imaging with PSMA-PET/CT can guide highly focused therapies, such as stereotactic body radiation therapy, to specific regions of concern. - PSMA-PET is indispensable for guiding MDT in patients with oligometastatic prostate cancer, which can lead to favourable disease-free and overall survival outcomes, particularly for bone disease in metachronous settings.
	**Optimising radiotherapy and surgical approaches:** - PSMA-PET plays a significant role in refining radiation prescriptions by improving target identification and dose management for nodal and metastatic sites, enabling “precision radiotherapy” and dose escalations. PSMA-PET findings have informed the development of guidelines for pelvic lymph node contouring. - The detailed anatomical and functional information from PSMA-PET, with or without prostate MRI (for dominant tumour location), allows for a more personalised approach to local prostate cancer management. - Imaging findings from PSMA-PET have been shown to lead to changes in management (including treatment intent, modality, or delivery) in a significant proportion of patients.
	**Theranostics patient selection**: - PSMA-PET is used for patient selection for PSMA-targeted radioligand therapy, as it can visualise PSMA-positive lesions. Before deciding on radioligand therapy, contrast-enhanced CT, WB-MRI or FDG-PET can be considered to detect the presence and extent of PSMA-negative disease.
Disease monitoring and response assessment	**Detection of biochemical recurrence or failure:** - PSMA-PET excels in detecting the location of BCR after primary curative treatment, even at low PSA levels, outperforming conventional imaging methods. MRI is also practical for detecting local recurrent cancer after primary therapy. - WB-MRI is less effective for detecting nodal disease recurrence.
	**Monitoring treatment response:** - PSMA-PET and WB-MRI are promising for tracking disease progression in suboptimal PSA responders in mHSPC and evaluating treatment response in mCRPC, where PSA response assessment becomes less reliable. - WB-MRI provides quantitative insights into tumour and host responses to effective therapies, especially in bone metastases (better than bone scan and CT). - PSMA-PET can be utilised to assess the response to RLT, indicating whether the therapy suppresses viable tumours or if resistant disease is emerging before clinical or biochemical failure becomes apparent.

*BCR* biochemical recurrence, *CT* computed tomography, *FDG* fluorodeoxyglucose, *MDT* metastasis-directed therapy, *MRI* magnetic resonance imaging, *PCa* prostate cancer, *PET* positron emission tomography, *PPP* PSMA PET progression, *PSA* prostate-specific antigen, *PSMA* prostate-specific membrane antigen, *PSMA-TV*  PSMA-derived tumour volume, *RLT* radioligand therapy, *RECIP* response evaluation criteria in PSMA PET/CT, *TL-PSMA* total lesion PSMA, *WB-MRI* whole-body magnetic resonance imaging

## Abbreviations

ADC: Apparent diffusion coefficient
ADT: Androgen deprivation therapy
APC: Advanced prostate cancer
ARPI: Androgen receptor pathway inhibitor
ASCO: American Society of Clinical Oncology
BS-CT: Bone scan and computed tomography
CT: Computed tomography
DWI: Diffusion-weighted imaging
EANM: European Association of Nuclear Medicine
EAU: European Association of Urology
HU: Hounsfield Units
mCRPC: Metastatic castration-resistant prostate cancer
MDT: Metastasis-directed treatment
MET-RADS-P: METastasis Reporting and Data System for Prostate Cancer
mHSPC: Metastatic hormone-sensitive prostate cancer
NCCN: National Comprehensive Cancer Network
OS: Overall survival
PCa: Prostate cancer
PCWG 3: Prostate Cancer Working Group 3
PET: Positron emission tomography
PPP: PSMA PET Progression
PROMISE: Prostate Cancer Molecular Imaging Standardized Evaluation
PSA: Prostate-specific antigen
PSMA: Prostate-specific membrane antigen
RAC: Response assessment category
RECIP: Response Evaluation Criteria in PSMA PET/CT
RECIST: Response Evaluation Criteria in Solid Tumours
rFF: Relative fat fraction
rPFS: Radiographic progression-free survival
SNMMI: Society of Nuclear Medicine and Molecular Imaging
SUVmax: Maximum standardised uptake value
TTV: Total tumour volume
WB-MRI: Whole-body magnetic resonance imaging
